# The Uric Acid to Albumin Ratio Predicts All-cause and Cardiovascular Mortality Among U.S. Adults Results from the National Health and Nutrition Examination Survey in 2003-2018

**DOI:** 10.7150/ijms.106664

**Published:** 2025-04-22

**Authors:** Guangyu Wang, Guangyu Li, Pengfei Wang, Minhua Zang, Jun Pu

**Affiliations:** Department of Cardiology, School of Medicine, Renji Hospital, Shanghai Jiao Tong University, Shanghai, China.

**Keywords:** Uric acid to albumin ratio, Mortality, Cardiovascular disease, NHANES

## Abstract

**Background:** The association between the uric acid to albumin ratio (UAR) and mortality in the general population remains poorly understood. This study aimed to investigate the associations of UAR with all-cause and cardiovascular mortality among American adults.

**Methods:** The study population comprised 19190 U.S. adults from the National Health and Nutrition Examination Survey (NHANES) conducted between 2003 and 2018. Mortality outcomes were ascertained through linkage to National Death Index (NDI) records, with follow-up extending to December 31, 2019. Multivariate Cox proportional hazards regression models with restricted cubic splines and trend analyses were utilized to assess the association between UAR and both all-cause and cardiovascular mortality. Subgroup analyses were conducted to assess whether the association between UAR and mortality varied across different demographic and clinical groups.

**Results:** During a median follow-up period of 98 months, 2296 all-cause deaths were recorded, including 597 deaths related to cardiovascular disease (CVD). After multivariable adjustment, no linear trends were observed between UAR and either all-cause or CVD mortality. Kaplan-Meier curves revealed a significant increase in both all-cause and CVD mortality with associated with higher UAR levels (p for log-rank test < 0.001 for both). Restricted cubic spline models indicated a J-shaped nonlinear association between UAR and both all-cause and CVD mortality, with inflection points at UAR levels of 1.40 for all-cause mortality and 1.88 for CVD mortality. Specifically, UAR values exceeding these inflection points were positively associated with mortality (HR 2.11, 95% CI = 1.74-2.55 for all-cause mortality; HR 5.21, 95% CI = 3.06-8.87 for CVD mortality). Conversely, UAR values below the inflection points were inversely associated with all-cause mortality (HR 0.68, 95% CI = 0.50-0.93) but not significantly associated with CVD mortality (HR 1.07, 95% CI = 0.73-1.58). This association remained consistent across subgroup analyses stratified by sex, age, race, diabetes, hypertension, BMI, and smoking status, with no significant interactions between these characteristics and UAR (p for interaction > 0.05).

**Conclusion:** This study identified a significant association between the UAR and both all-cause and CVD mortality in the general population. A J-shaped nonlinear association was observed, with inflection points at UAR levels of 1.40 for all-cause mortality and 1.88 for CVD mortality.

## Introduction

Despite decades of medical advancements, cardiovascular disease (CVD) remains the leading global cause of mortality, representing a staggering and avoidable global health crisis 1, 2. Although the global age-standardized CVD mortality rate has declined, the absolute number of CVD-related deaths has surged dramatically—from 12.4 million in 1990 to a heartbreaking 19.8 million in 2022 1. Even more concerning, 34% of these deaths occur before the age of 70, robbing individuals of decades of life and placing a heavy burden on healthcare systems worldwide 1. This stark reality underscores the critical need to identify and address modifiable risk factors to prevent premature mortality and improve long-term health outcomes.

Uric acid (UA), a byproduct of purine metabolism, has emerged as a key contributor to the development of atherosclerosis and is increasingly recognized for its role in predicting adverse outcomes in coronary artery disease (CAD) at elevated levels 3-6. UA is not merely a risk marker; it actively modulates endothelial dysfunction, inflammation, and vascular disease—core drivers of CVD progression 7. Numerous studies have established a robust association between elevated UA levels and increased mortality among patients with CVD or diabetes 4, 8. Meanwhile, albumin (Alb), the predominant circulating protein in human serum, plays a critical role in maintaining physiological balance 9, with low levels correlating with worse CAD severity and higher mortality rates 10-14. However, these two markers have traditionally been studied in isolation, leaving a substantial gap in our understanding of how their combined effects might provide a more comprehensive measure of CVD risk.

We propose that integrating UA and Alb into a single metric—the uric acid to albumin ratio (UAR)—reflects multidimensional physiological dysfunction associated with inflammation, oxidative stress, and nutrition 5, 15. This approach may yield a deeper understanding of their interplay and implications for CVD risk. Although UAR has already shown promise in specific populations such as those with unstable angina 16, acute myocardial infarction 17, and aortic dissection 18, its potential as a predictive tool for all-cause and CVD mortality in the general population remains underexplored.

Our study aims to bridge this gap, using a large, nationally representative sample from the National Health and Nutrition Examination Survey (NHANES) cohort (2003-2018) to rigorously assess the association between UAR and mortality outcomes. Identifying UAR as a potential biomarker, we have the opportunity to transform clinical practice, allowing for earlier, more precise interventions that could dramatically mitigate this pressing public health issue.

## Methods

### Study design and population

**Figure 1:**
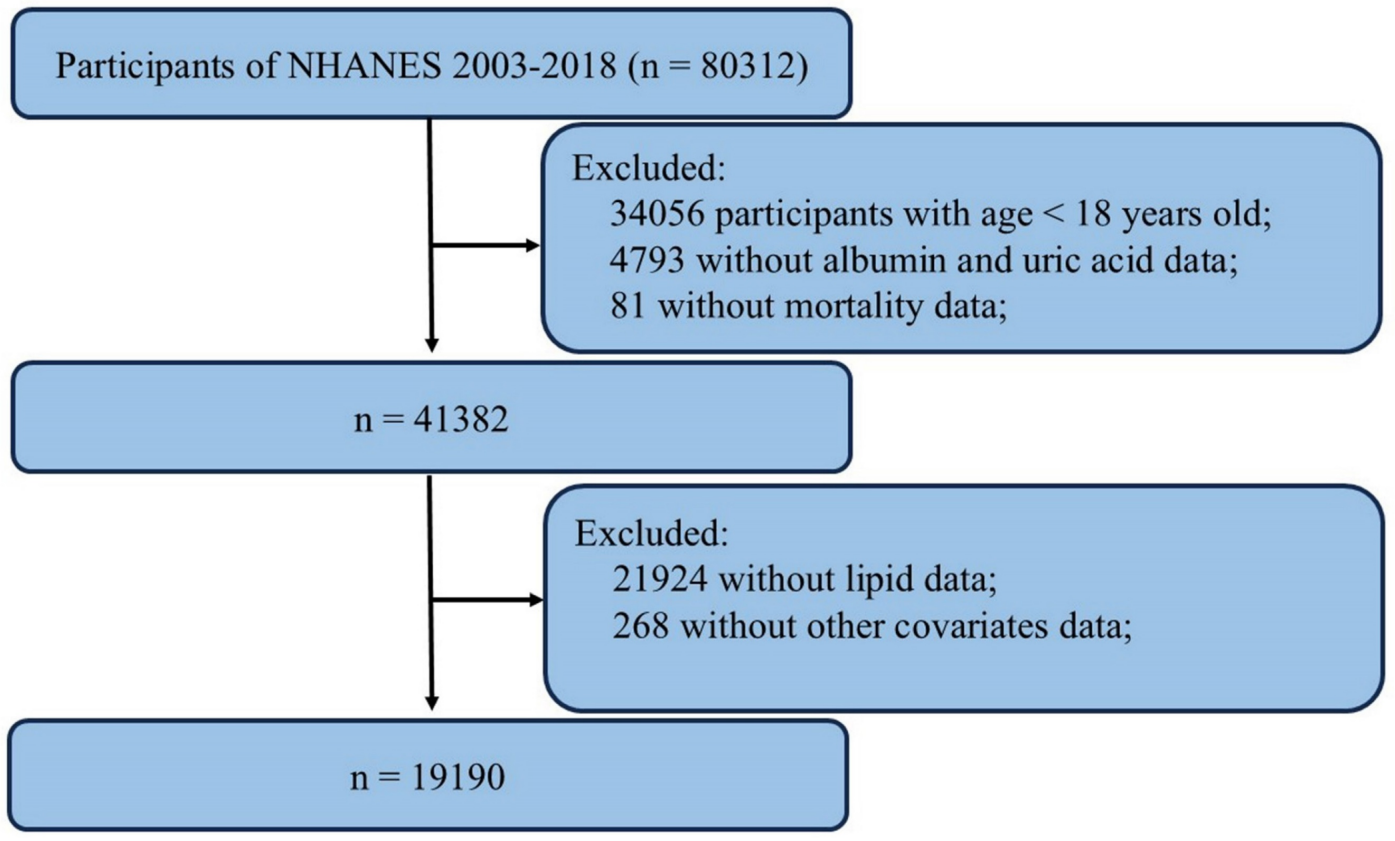
Flowchart of the sample selection from the 2003-2018 NHANES.

The data utilized in this study were obtained from the National Health and Nutrition Examination Survey (NHANES), a publicly available national survey program administered by the National Center for Health Statistics (NCHS). NHANES employs a stratified, multistage probability sampling method to select individuals from the general population, ensuring representation of the civilian noninstitutionalized resident population. The survey includes interviews covering demographic, socioeconomic, dietary, and health-related factors. Data from NHANES are utilized in epidemiological studies and health sciences research and are accessible to the public via the NHANES website. Detailed information regarding study design, survey methods, population characteristics, and data is available on the NHANES website (https://www.cdc.gov/nchs/nhanes/). The NHANES protocol received approval from the NCHS Research Ethics Review Board, and written informed consent was obtained from all participants. We compiled data spanning 8 cycles from NHANES (2003-2004, 2005-2006, 2007-2008, 2009-2010, 2011-2012, 2013-2014, 2015-2016, 2017-2018). A total of 80312 samples were enrolled at first. Then, 19190 participants were selected as the final analysis sample after excluding people with age < 18 years old (n = 34056), missing data on albumin and uric acid (n = 4793), missing data on mortality (n = 81), incomplete lipids (n= 21924) and other covariates data (n = 268) (**Figure 1**).

### Measurement of UAR

The uric acid to albumin ratio was calculated by dividing the uric acid (mg/dl) by the albumin (g/dl) value, both obtained from laboratory tests. Blood specimens were collected following established venipuncture protocols and procedures. Uric acid measurements were performed using a Beckman Synchron LX20 in NHANES 2003-2007, a Beckman UniCel DxC800 Synchron in NHANES 2008-2016, and a Roche Cobas 6000 analyzer in NHANES 2017-2018. In this method, uric acid is oxidized by uricase. The peroxide produced from this reaction is then acted upon by peroxidase in the presence of 4 aminophenazone, producing a measurable colored product. This is a two-point, endpoint reaction, with measurements taken at 546 nm (secondary wavelength 700 nm). Albumin measurements were performed using a Beckman Synchron LX20 in NHANES 2003-2007, a Beckman UniCel DxC800 Synchron in NHANES 2008-2016, and a Roche Cobas 6000 (c501 module) analyzer in NHANES 2017-2018. The method for measuring albumin concentration utilizes bromcresol purple (BCP) dye. When the dye selectively binds with albumin in a pH range of 5.2-6.8, a color change occurs, which is measured at 600 nm. The secondary wavelength was 700 nm. This is a two-point, endpoint reaction specific to albumin.

### Assessment of covariates

Covariates were collected through standardized interviews, physical and laboratory examinations, and questionnaires administered by well-trained medical personnel. These covariates encompassed demographic characteristics (age, gender, race/ethnicity, education levels and ratio of family income to poverty [PIR]), medical history (hypertension and diabetes mellitus), lifestyle (smoking status, alcohol intake, and body mass index [BMI]) and laboratory results (uric acid, albumin, low-density lipoprotein cholesterol [LDL-c], high-density lipoprotein cholesterol [HDL-c] and total cholesterol [TC], triglyceride [TG], alanine aminotransferase [ALT], aspartate aminotransferase [AST], glycohemoglobin [HbA1C]). Furthermore, we computed the estimated glomerular filtration rate (eGFR) using the Chronic Kidney Disease Epidemiology Collaborative equation to assess the participants' kidney function. Race/ethnicity was categorized into five groups: Mexican American, other Hispanic, non-Hispanic white, non-Hispanic black and other race. Educational levels were categorized into five groups: <9th grade, 9-11th grade, high school, college and graduate or above. Participants were defined as alcohol users if they had consumed at least 4 drinks/day. Smoking status was categorized as every day, some day, and not at all. Self-reported personal interview data provided the medical and medication history of hypertension and diabetes mellitus. BMI was calculated as the weight in kilograms divided by the square of height in meters, which was obtained from the body measurements. BMI was categorized as < 25, 25-29.9, and ≥ 30 kg/m^2^.

During the personal interview, participants were administered a standardized medical condition questionnaire addressing various health issues, such as congestive heart failure (CHF), coronary heart disease (CHD). Participants were asked the following question during the interview: "Has a doctor or other health professional ever informed you that you have: CHF/CHD?" (this constituted a set of five questions with identical phrasing). Participant who answered "yes" to the preceding questions was classified as having CHF/CHD.

### Ascertainment of mortality

To determine mortality status in the follow-up population, we utilized the NHANES public-use linked mortality file as of December 31, 2019. This file was linked to the National Death Index (NDI) by the NCHS using a probability matching algorithm. The follow-up began on the interview date and ended on the date of death or at the conclusion of the mortality tracking period (December 31, 2019). Additionally, we used the International Statistical Classification of Diseases, 10th Revision (ICD-10) to identify disease-specific deaths, with the NCHS classifying heart diseases (054-064), malignant neoplasms (019-043), and all other causes (010) for our study.

### Statistical analysis

Statistical analysis adhered to Centers for Disease Control guidelines, applying NHANES sampling weights to account for the complex multistage cluster survey design. Continuous variables were presented as the means with standard error (SE), and categorical variables as percentages. Differences across UAR quartiles were assessed using weighted linear regression for continuous variables and weighted chi-square test for categorical variables. Multivariate Cox regression models were employed to examine the relationship between UAR and mortality across three models. Model 1 was unadjusted; Model 2 was adjusted for age, gender and race. Model 3 was adjusted for age, gender, race, education level, marital status, PIR, BMI, smoking status, drinking status, hypertension, diabetes, CHF, CHD, HDL, LDL, TG, TC, ALT, AST, HbA1c, and eGFR. Cox proportional hazards regression models, incorporating restricted cubic splines and smooth curve fitting (penalized spline method), were used to explore nonlinear relationship between UAR with mortality. In cases of a nonlinear relationship, a recursive algorithm was used to identify inflection points between UAR and both all-cause and CVD mortality. A two-segment Cox proportional hazards model was then applied to both sides of the inflection point to assess the association between UAR and mortality risk. Subgroup analyses were performed, stratifying by age (< 60 years old or ≥ 60 years old), gender, race, BMI (< 25, 25-29.9, and ≥ 30), history of diabetes mellitus or hypertension, and smoking status, with adjustments made for all covariates in the regression models. Additionally, an interaction term was included to evaluate the heterogeneity of associations among subgroups. Statistical significance was defined as p < 0.05. Statistical analyses were performed using Empower software (www.empowerstats.com; X&Y solutions, Inc., Boston MA) and R version 4.2.0 (http://www.R-project.org, The R Foundation).

## Results

### Baseline characteristics

**Table 1** presents the weighted baseline characteristics of the study participants. Our analysis included 19190 participants, with a mean age of 46.64 ± 0.24 years, of whom 47.90% were men and 52.10% were women. The weighted mean UAR was 1.29 ± 0.01, with quartiles ranging from 0.09-1.06, 1.07-1.27, 1.28-1.51 and 1.52 ± 4.39, respectively. Age, gender, race, education level, marital status, BMI, smoker status, diabetes mellitus, hypertension, alcohol user, TG, TC, HDL-c, LDL-c, HbA1c, AST, ALT, albumin, eGFR, CHF, CHD, hypotensive agent, all-cause mortality and CVD mortality differed significantly across UAR quartiles (all p < 0.05). No significant differences were observed in PIR or the use of hypoglycemic agent (all p > 0.05).

### Kaplan-Meier survival analysis curves for mortality

**Figure 2:**
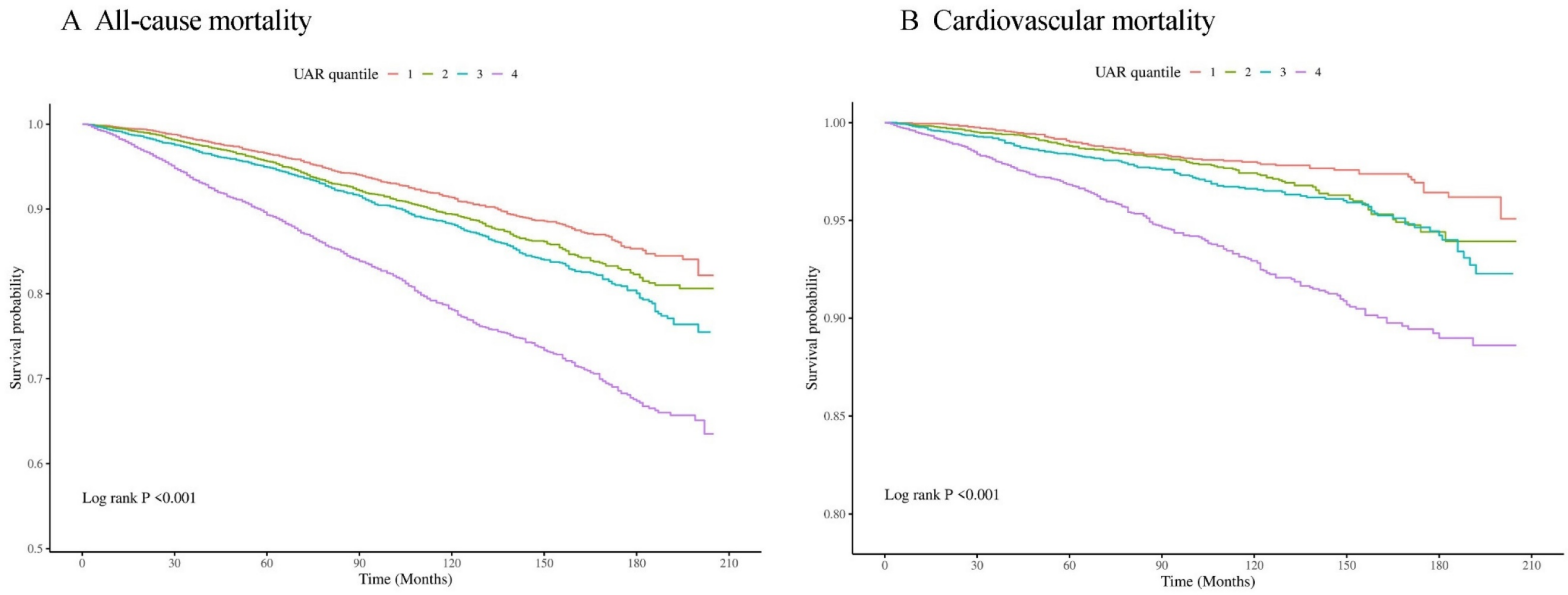
Kaplan-Meier survival analysis curves for all-cause (A) and cardiovascular mortality (B).

During the 98-month follow-up period, there were 2296 incident cases of all-cause mortality and 597 incident cases of CVD mortality. Kaplan-Meier analysis revealed a significant difference in mortality among these groups within the overall populations (all p for log-rank test < 0.001) (**Figure 2**).

### Association between UAR and mortality

We constructed three Cox regression models to investigate the independent association between UAR and mortality risk. In the Model 1 and 2, we identified that the risk for all-cause and CVD mortality significantly increased as UAR increased (**Table 2**). After adjusting for age, gender, race, education level, marital status, PIR, BMI, smoking status, drinking status, hypertension, diabetes, CHF, CHD, HDL, LDL, TG, TC, ALT, AST, HbA1c, and eGFR in Model 3, the multivariate-adjusted hazard ratios (HRs) and 95% confidence intervals (CIs) from the lowest to the highest UAR quartiles were 1.00 (reference), 0.83 (0.65, 1.05), 0.78 (0.62, 0.99), and 1.01 (0.80, 1.28), respectively, for all-cause mortality (p for trend = 0.07); and 1.00 (reference), 0.91 (0.62, 1.32), 0.77 (0.53, 1.12), and 1.02 (0.75, 1.40), respectively, for CVD mortality (p for trend = 0.21) (**Table 2**).

### The detection of nonlinear relationships

**Figure 3:**
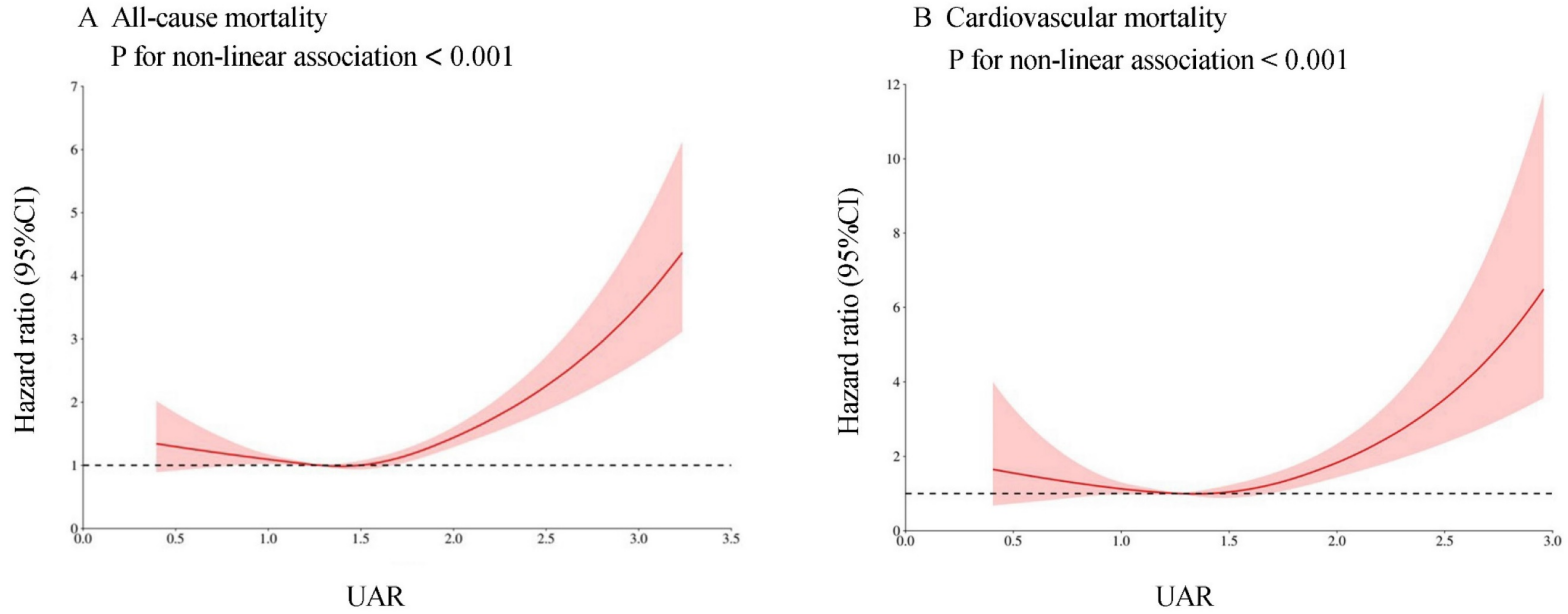
Association between UAR and all-cause (A) and cardiovascular mortality (B) in the general population. Adjusted for age, gender, race, education level, marital status, PIR, BMI, smoking status, drinking status, hypertension, diabetes, CHF, CHD, HDL, LDL, TG, TC, ALT, AST, HbA1c, and eGFR. The solid line and red area represent the estimated values and their corresponding 95% CIs, respectively.

Cox proportional hazards regression models with restricted cubic splines and smooth curve fitting (penalized spline method) indicated a nonlinear relationship between UAR and the risk of all-cause and CVD mortality. We employed restricted cubic spline analysis to further investigate this association. We discovered J-shaped associations between the UAR and all-cause (**Figure 3A**) and CVD mortality (**Figure 3B**). We combined a Cox proportional hazards model with a two-piecewise Cox proportional hazards model to investigate the nonlinear relationship between the UAR and both all-cause and CVD mortality. Based on the two-piecewise Cox proportional hazards model, we identified the inflection points for all-cause and CVD mortality as 1.40 and 1.88, respectively (both p values for the log-likelihood ratio < 0.05) (**Table 3**). When the UAR was greater than or equal to 1.40 or 1.88, a 1-unit increase in UAR was associated with a 2.11-fold and 5.21-fold greater risk of all-cause and CVD mortality, respectively (HR 2.11, 95% CI 1.74-2.55 and HR 5.21, 95% CI 3.06-8.87, respectively). When the UAR was less than 1.40, a 1-unit decrease in UAR level was associated with a 32% greater risk of all-cause mortality (HR 0.68, 95% CI 0.50-0.93). However, when the UAR was less than 1.88, there was no significant association with CVD mortality (HR 1.07, 95% CI 0.73-1.58).

### Stratified analysis

**Figure 4:**
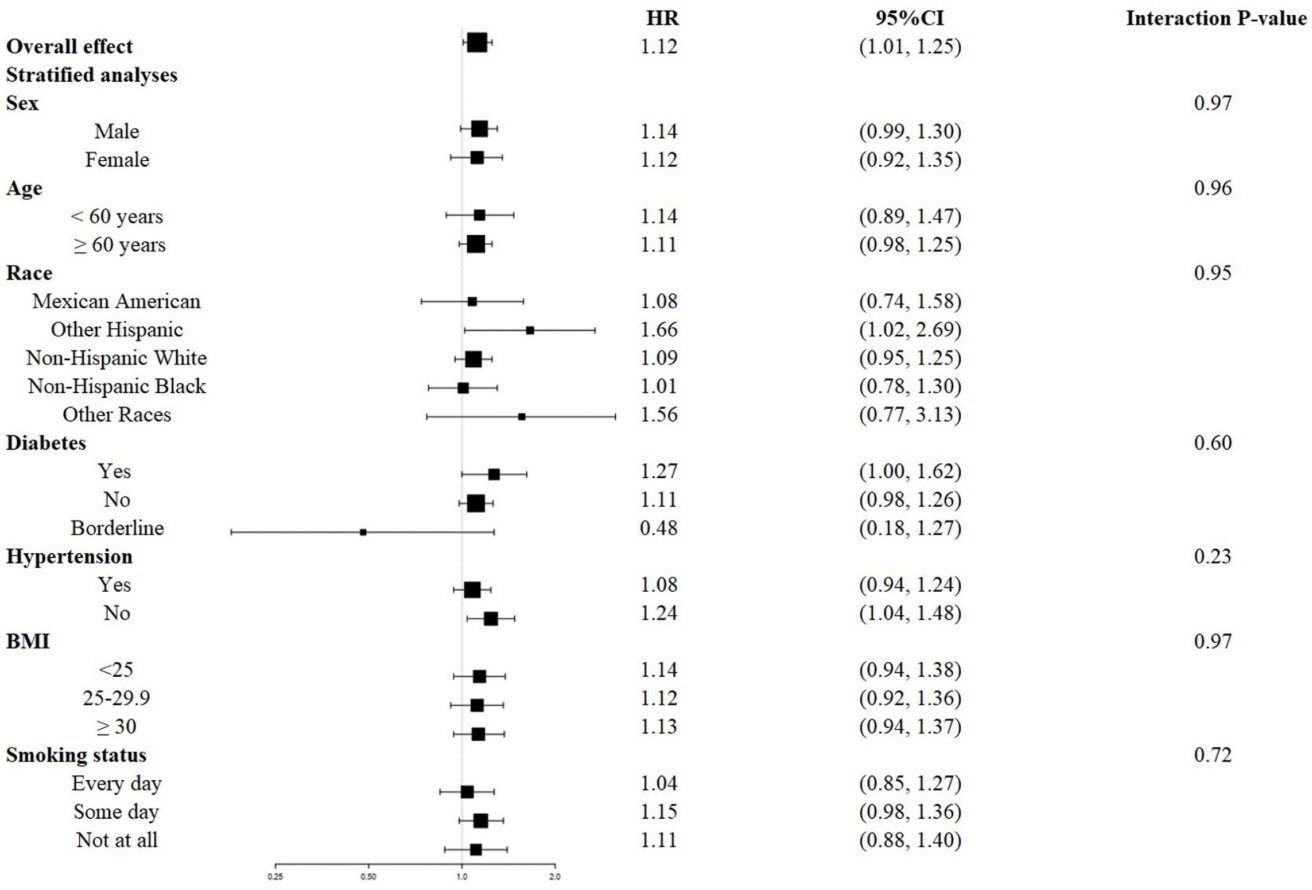
Forest plots of stratified analyses of UAR and all-cause mortality. Age, gender, race, education level, marital status, PIR, BMI, smoking status, drinking status, hypertension, diabetes, CHF, CHD, HDL, LDL, TG, TC, ALT, AST, HbA1c, and eGFR were all adjusted except the variable itself.

The survival disadvantage associated with a higher UAR (≥ 1.40 for all-cause mortality and ≥ 1.88 for CVD mortality) compared to a lower UAR (< 1.40 for all-cause mortality and < 1.88 for CVD mortality) was consistent across subgroups stratified by age, gender, race, BMI, history of diabetes, history of hypertension, and smoking status (**Figures 4** and** 5**). No significant interactions were observed between the UAR and the stratified variables. Moreover, our findings indicated a stronger positive association between the UAR and all-cause mortality in Other Hispanics, patients with a history of diabetes, and individuals without a history of hypertension, although the interaction tests did not reach significance. Furthermore, a stronger positive association between UAR and CVD mortality was observed in Non-Hispanic Whites, Other Races, those with a history of hypertension, individuals with a BMI ≥ 30 kg/m^2^, and non-smokers, although the interaction tests were also not significant.

## Discussion

To the best of our knowledge, this study is the first to reveal a J-shaped association between UAR and both all-cause and CVD mortality in the general population. Our threshold effect analysis identified critical turning points at UAR levels of 1.40 for all-cause mortality and 1.88 for CVD mortality, underscoring the robustness of UAR as a significant predictor of mortality. These findings highlight the potential utility of UAR as a novel and promising biomarker for identifying individuals at high risk of mortality, providing valuable insights for clinical practice.

Previous studies have investigated the association between UAR and mortality within specific populations, including those with unstable angina pectoris 16, acute myocardial infarction 17, acute kidney injury 19, and acute type A aortic dissection 18. For example, Kalkan et al. analyzed 4599 patients diagnosed with ST-elevation myocardial infarction who underwent percutaneous intervention between 2015 and 2020, reporting that UAR was a significant predictor of mortality (HR 1.33, 95% CI = 1.16-1.52) 17. Similarly, Wang et al. studied 289 patients with acute type A aortic dissection from the Cardiovascular Surgery Department of the First Affiliated Hospital of Xi'an Jiaotong University between January 2019 and September 2020, finding that preoperative UAR was an independent risk factor for one-year mortality (HR 1.90, 95% CI = 1.10-3.31) 18. Furthermore, UAR was shown to be a superior predictor of one-year mortality compared to either UA or Alb alone 18. In another study, Li et al. enrolled 2298 patients with unstable angina pectoris from the Cardiovascular Department of the Beijing Friendship Hospital between January 2013 and December 2018, revealing that both cardiac and all-cause mortality rates were significantly higher in the high UAR group (≥ 8.38) than in the low UAR group (< 8.38, p = 0.007, and p < 0.001) 16. Notably, UAR was independently associated with long-term cardiac mortality (HR 1.26, 95% CI = 1.08-1.46) in multivariate Cox regression analysis 16. However, these findings were limited in scope, often focusing on narrow, disease-specific cohorts. Our study breaks new ground by applying this analysis to the general population, addressing a critical gap in the literature and broadening the potential clinical utility of UAR as a reliable mortality predictor.

In our study, individuals with a UAR ≤ 1.06 exhibited lower rates of all-cause and CVD mortality compared to those with a UAR > 1.06. Although the precise biological mechanisms underlying the association between elevated UAR and mortality risk remain unclear, key pathways likely involve inflammation and nutritional status, both of which play crucial roles in determining mortality risk 20, 21. UA participates in various pathological processes, including endothelial dysfunction, oxidative stress, systemic inflammation, and activation of renin-angiotensin system 22, all of which contribute to further endothelial dysfunction and vascular smooth muscle cell proliferation. On the other hand, Alb serves as a key marker of both inflammation severity and nutritional status 15, 23. Previous studies have demonstrated that elevated UA and reduced Alb are associated with increased mortality risk across several disease-specific populations, such as those with diabetes 8, 24, acute myocardial infarction 25, 26, heart failure 27, and ischemic stroke 28. However, these studies did not integrate both markers, which could yield additional insights. Our study found that elevated UAR correlates with an increased risk of all-cause and CVD mortality in the general population, indicating the necessity of future research to elucidate the mechanisms by which UAR influences mortality risk.

Interestingly, our findings also indicate a J-shaped association between UAR and both all-cause and CVD mortality in the general population. Lower UAR levels (specifically UAR < 1.40 for all-cause mortality) significantly modified the relationship between UAR and the risk of all-cause mortality. After adjusting for potential confounders, each unit decrease in UAR was associated with a 32% increase in the risk of all-cause mortality among participants with UAR levels below this threshold. While UA possesses antioxidant properties, hypouricemia has been shown to damage the endothelium and trigger oxidative stress-related disease such as hypertension, diabetes, and kidney disease 29, 30. Studies have also indicated that lower UA levels are significantly associated with increased all-cause and CVD mortality in various patient populations 31, 32. Additionally, prospective cohort studies have indicated a U-shaped relationship between UA levels and mortality in the general population 33, 34, suggesting that both low and high UA levels are linked to an increased risk of mortality. Therefore, maintaining an optimal UAR level is crucial, as both low and high levels may lead to detrimental health consequences. Our study opens the door for future research aimed at understanding the precise mechanisms by which UAR influences mortality risk, potentially leading to new treatments and strategies that could save lives.

Identifying high-risk individuals for all-cause and CVD mortality in the general population is crucial for public health interventions 1. Our stratified analysis revealed a significantly increased risk of all-cause mortality linked to elevated UAR (≥ 1.40), particularly among individuals with diabetes, without hypertension, and within the Other Hispanic population. Previous studies have shown that the prevalence of diabetes and hypertension is higher in groups with adverse outcomes and elevated UAR levels 35-37, suggesting that these conditions combined with higher UAR increase susceptibility to adverse events. However, these findings differ somewhat from ours, potentially due to differences in sample size, follow-up duration, and characteristics of the target population. Additionally, we observed an increased risk of CVD mortality associated with elevated UAR (≥ 1.88), particularly among individuals with hypertension, BMI ≥ 30, non-smokers, and the Non-Hispanic White population. Previous research has identified a positive association between UAR and the non-dipper hypertension pattern in hypertensive patients 38, 39, as well as obesity in adolescents, both of which are known to increase CVD mortality risk 40-42. While most prior studies have been conducted in Asia and Europe 16-18, 36, 37, limited research has focused on North America populations, particularly in diverse ethnic subgroups. These findings not only underscore the importance of early intervention in high-risk groups but also provide crucial insights for public health initiatives aimed at reducing mortality in vulnerable populations.

### Strengths and limitations

This study possesses several notable strengths. First, our research utilized data from NHANES, a comprehensive, population-based sample that adheres to standardized protocols. Second, our adjustment for confounding variables was informed by previous research examining the relationship between UAR and mortality, enhancing the reliability of our findings. Finally, we are the first to illustrate the relationship between UAR and mortality using the restricted cubic spline model, which adds a novel analytical perspective to our results.

However, several limitations must be acknowledged. The cross-sectional study design of our study precludes establishing a causal relationship between UAR and mortality risk. Furthermore, UAR was measured only at baseline, which may not fully capture the time-dependent dynamics of UAR and its association with mortality risk. Despite our rigorous efforts to adjust for confounding variables, we cannot entirely eliminate the potential residual confounding effects from unmeasured or excluded variables. Lastly, as our data are derived from a U.S. database, this may limit generalizability of our findings to other regions or populations. Therefore, additional epidemiological studies are warranted to further establish the predictive value of UAR in clinical practice.

## Conclusions

This study demonstrates that UAR is a robust and independent predictor of both all-cause and cardiovascular mortality in the general population, with the association following a distinct nonlinear pattern. Given the strong and consistent relationship between UAR and mortality risk, UAR emerges as a highly promising biomarker that could revolutionize how clinicians identify individuals at elevated risk. However, to fully harness its potential and integrate it into clinical practice, further research is crucial to validate and expand upon these findings, ensuring that UAR becomes a reliable tool in mortality risk assessment.

## Figures and Tables

**Figure 5:**
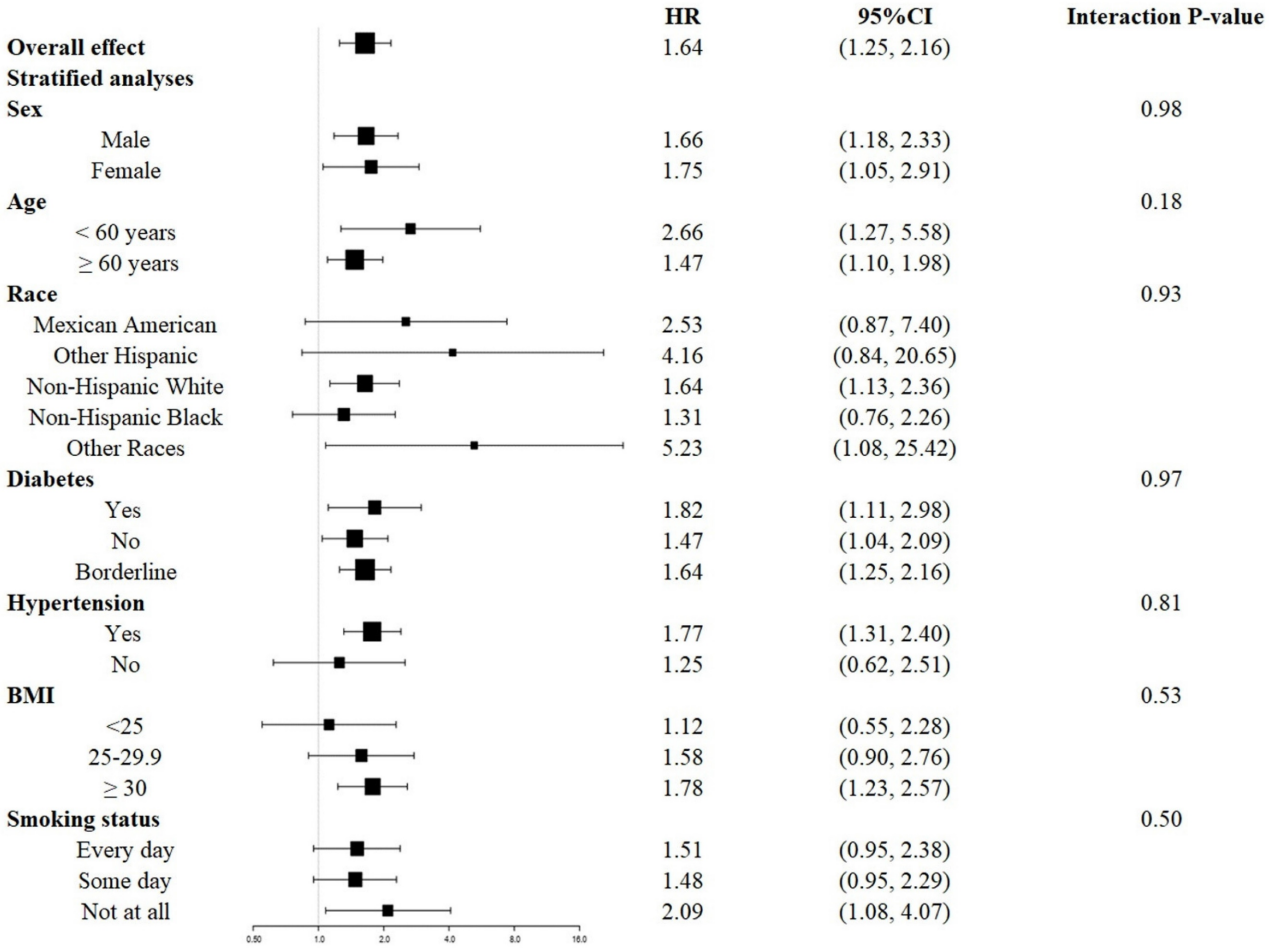
Forest plots of stratified analyses of UAR and cardiovascular mortality. Age, gender, race, education level, marital status, PIR, BMI, smoking status, drinking status, hypertension, diabetes, CHF, CHD, HDL, LDL, TG, TC, ALT, AST, HbA1c, and eGFR were all adjusted except the variable itself.

**Table 1:** Weighted baseline characteristics of participants according to UAR quartiles

	Overall	Q1 (0.09-1.06)	Q2 (1.07-1.27)	Q3 (1.28-1.51)	Q4 (1.52-4.39)	P-value
	n = 19190	n = 4796	n = 4771	n = 4815	n = 4808	
Age (year)	46.64 ± 0.24	43.79 ± 0.33	45.20 ± 0.35	47.34 ± 0.39	50.57 ± 0.35	< 0.0001
**Gender, % (SE)**						< 0.0001
Male	47.90 (0.37)	19.53 (0.79)	45.47 (0.90)	60.74 (0.81)	67.60 (0.89)	
Female	52.10 (0.37)	80.47 (0.79)	54.53 (0.90)	39.26 (0.81)	32.40 (0.89)	
**Race, % (SE)**						< 0.0001
Mexican American	8.56 (0.64)	9.68 (0.75)	9.23 (0.74)	8.27 (0.71)	6.90 (0.67)	
Other Hispanic	5.34 (0.43)	6.55 (0.59)	5.25 (0.51)	5.08 (0.52)	4.39 (0.42)	
Non-Hispanic White	67.31 (1.20)	66.07 (1.38)	66.38 (1.35)	68.38 (1.41)	68.47 (1.43)	
Non-Hispanic Black	11.34 (0.66)	10.20 (0.75)	11.14 (0.70)	10.54 (0.66)	13.71 (0.96)	
Other Races	7.46 (0.38)	7.51 (0.54)	8.00 (0.52)	7.72 (0.54)	6.53 (0.50)	
**Education level, % (SE)**						< 0.0001
Less than 9th grade	5.71 (0.29)	6.07 (0.47)	5.54 (0.37)	5.32 (0.37)	5.95 (0.37)	
9-11th grade	10.83 (0.46)	9.71 (0.59)	11.10 (0.68)	11.64 (0.77)	10.89 (0.59)	
High school graduate	23.55 (0.57)	21.41 (0.92)	23.22 (0.95)	24.37 (0.97)	25.34 (1.01)	
College degree	30.94 (0.61)	30.85 (1.05)	29.39 (0.94)	31.05 (0.98)	32.55 (1.01)	
College and above	28.93 (0.94)	31.95 (1.45)	30.71 (1.12)	27.60 (1.33)	25.19 (1.05)	
**Marital status, % (SE)**						< 0.0001
Married	55.78 (0.74)	55.29 (0.94)	53.88 (1.19)	57.09 (1.07)	56.90 (1.14)	
Widowed	5.48 (0.21)	4.64 (0.36)	4.93 (0.37)	5.05 (0.37)	7.49 (0.45)	
Divorced	10.08 (0.34)	10.11 (0.74)	9.87 (0.57)	10.07 (0.54)	10.28 (0.61)	
Separated	2.35 (0.16)	2.78 (0.29)	2.31 (0.26)	2.18 (0.25)	2.12 (0.24)	
Never married	17.99 (0.57)	18.43 (0.83)	19.58 (0.96)	17.43 (0.93)	16.43 (0.80)	
Living with partner	8.28 (0.36)	8.68 (0.56)	9.43 (0.69)	8.18 (0.62)	6.73 (0.48)	
PIR	2.97 ± 0.03	2.92 ± 0.05	2.98 ± 0.04	3.00 ± 0.05	2.99 ± 0.04	0.3268
**Smoking status, % (SE)**						< 0.0001
Every day	54.91 (0.69)	59.81 (1.30)	56.66 (0.98)	52.51 (1.33)	50.35 (1.13)	
Some day	24.96 (0.57)	19.86 (0.93)	22.35 (0.83)	26.45 (1.03)	31.70 (1.09)	
Not at all	20.12 (0.57)	20.33 (1.00)	20.98 (0.89)	21.04 (0.97)	17.95 (0.72)	
**Diabetes mellitus, % (SE)**						< 0.0001
Yes	8.74 (0.30)	6.10 (0.39)	7.96 (0.58)	7.69 (0.50)	13.67 (0.62)	
No	89.26 (0.33)	92.68 (0.47)	90.40 (0.61)	90.02 (0.57)	83.41 (0.65)	
Borderline	1.95 (0.14)	1.16 (0.24)	1.63 (0.24)	2.18 (0.25)	2.91 (0.31)	
**Hypertension, % (SE)**						0.0002
Yes	31.50 (0.59)	20.16 (0.85)	26.15 (0.83)	33.27 (0.92)	47.81 (1.09)	
No	69.39 (0.59)	79.82 (0.85)	73.77 (0.84)	66.49 (0.94)	52.09 (1.09)	
**Alcohol user, % (SE)**						< 0.0001
Yes	15.47 (0.43)	10.10 (0.72)	14.85 (0.75)	16.36 (0.76)	20.62 (0.91)	
No	84.47 (0.43)	89.73 (0.74)	85.13 (0.75)	83.60 (0.76)	79.35 (0.91)	
**BMI (kg/m2)**	28.87 ± 0.09	25.47 ± 0.11	27.83 ± 0.12	29.76 ± 0.13	32.79 ± 0.16	< 0.0001
**Laboratory parameters**						
TG (mmol/L)	1.33 ± 0.01	1.06 ± 0.01	1.25 ± 0.02	1.44 ± 0.02	1.59 ± 0.02	< 0.0001
TC (mmol/L)	4.97 ± 0.01	4.91 ± 0.02	4.95 ± 0.02	5.01 ± 0.02	5.01 ± 0.02	0.0020
HDL-c (mmol/L)	1.41 ± 0.01	1.59 ± 0.01	1.45 ± 0.01	1.34 ± 0.01	1.26 ± 0.01	< 0.0001
LDL-c (mmol/L)	2.95 ± 0.01	2.83 ± 0.02	2.93 ± 0.02	3.01 ± 0.02	3.01 ± 0.02	< 0.0001
HbA1c (%)	5.58 ± 0.01	5.49 ± 0.02	5.54 ± 0.02	5.57 ± 0.02	5.74 ± 0.02	< 0.0001
ALT (U/L)	23.88 ± 0.11	19.88 ± 0.18	22.85 ± 0.21	25.44 ± 0.23	27.68 ± 0.30	< 0.0001
AST (U/L)	24.09 ± 0.10	22.23 ± 0.14	23.52 ± 0.15	24.64 ± 0.19	26.16 ± 0.25	< 0.0001
Albumin (g/dL)	4.23 ± 0.01	4.28 ± 0.01	4.27 ± 0.01	4.23 ± 0.01	4.12 ± 0.01	< 0.0001
Uric acid (mg/dL)	5.44 ± 0.02	3.86 ± 0.01	4.99 ± 0.01	5.88 ± 0.01	7.21 ± 0.02	< 0.0001
eGFR (ml/min/1.73m^2^)	98.48 ± 0.33	105.14 ± 0.44	101.22 ± 0.49	97.07 ± 0.48	89.72 ± 0.54	< 0.0001
**CHF, % (SE)**						< 0.0001
Yes	2.39 (0.15)	0.93 (0.15)	1.47 (0.22)	2.09 (0.27)	5.31 (0.43)	
No	97.46 (0.15)	98.98 (0.15)	98.47 (0.22)	97.68 (0.29)	94.46 (0.42)	
**CHD, % (SE)**						< 0.0001
Yes	3.50 (0.21)	1.77 (0.26)	2.86 (0.30)	3.85 (0.40)	5.69 (0.47)	
No	96.25 (0.23)	98.13 (0.26)	96.98 (0.30)	95.77 (0.43)	93.95 (0.49)	
**Drug use**						
**Hypoglycemic agent, % (SE)**						0.3972
Yes	46.00 (1.29)	42.89 (2.56)	45.08 (2.68)	45.12 (2.45)	48.91 (2.11)	
No	53.83 (1.29)	56.92 (2.57)	54.64 (2.63)	54.88 (2.45)	50.91 (2.12)	
**Hypotensive agent, % (SE)**						0.0192
Yes	86.56 (0.80)	82.88 (1.95)	85.93 (1.25)	86.03 (1.35)	88.89 (1.01)	
No	13.43 (0.80)	17.04 (1.94)	14.07 (1.25)	13.97 (1.35)	11.11 (1.01)	
**Outcomes, % (SE)**						
All-cause mortality	8.25 (0.31)	5.95 (0.43)	6.70 (0.44)	7.72 (0.45)	13.08 (0.62)	< 0.0001
Cardiovascular mortality	2.08 (0.13)	1.23 (0.16)	1.69 (0.21)	1.91 (0.23)	3.62 (0.31)	< 0.0001

Values are presented as the mean ± standard error (SE) unless stated otherwise. BMI, body mass index; TG, triglyceride; TC, total cholesterol; HDL-c, high density lipoprotein cholesterol; LDL-c, low density lipoprotein cholesterol; HbA1C, glycohemoglobin; CHF, congestive heart failure; CHD, coronary heart disease; eGFR, estimated glomerular filtration rate; ALT, alanine aminotransferase; AST, aspartate aminotransferase.

**Table 2:** HRs (95%CIs) for mortality according to UAR quartiles in the general population from the NHANES 2003-2018 cohort

	UAR quartiles				
	Q1 (0.09-1.06)	Q2 (1.07-1.27)	Q3 (1.28-1.51)	Q4 (1.52-4.39)	P trend
**All-cause mortality**					
Number of deaths (%)	382 (7.96)	461 (9.66)	544 (11.30)	909 (18.91)	
Model 1	1.00	1.14 (0.95, 1.37)	1.31 (1.11, 1.54)	2.35 (2.00, 2.75)	< 0.01
HR (95% CI) P-value		0.16	< 0.01	< 0.01	
Model 2	1.00	0.90 (0.75, 1.09)	0.87 (0.72, 1.05)	1.22 (1.02, 1.44)	< 0.01
HR (95% CI) P-value		0.28	0.14	0.03	
Model 3	1.00	0.83 (0.65, 1.05)	0.78 (0.62, 0.99)	1.01 (0.80, 1.28)	0.07
HR (95% CI) P-value		0.12	0.04	0.92	
**CVD mortality**					
Number of deaths (%)	89 (1.86)	111 (2.33)	139 (2.89)	258 (5.37)	
Model 1	1.00	1.39 (0.98, 1.97)	1.62 (1.24, 2.11)	3.44 (2.71, 4.38)	< 0.01
HR (95% CI) P-value		0.07	< 0.01	< 0.01	
Model 2	1.00	1.01 (0.69, 1.48)	0.98 (0.66, 1.44)	1.51 (1.08, 2.12)	< 0.01
HR (95% CI) P-value		0.95	0.91	0.02	
Model 3	1.00	0.91 (0.62, 1.32)	0.77 (0.53, 1.12)	1.02 (0.75, 1.40)	0.21
HR (95% CI) P-value		0.60	0.17	0.88	

Model 1: Non-adjusted; Model 2: Adjusted for age, gender and race; Model 3: Adjusted for age, gender, race, education level, marital status, PIR, BMI, smoking status, drinking status, hypertension, diabetes, CHF, CHD, HDL, LDL, TG, TC, ALT, AST, HbA1c, and eGFR.

**Table 3:** Threshold effect analysis of UAR on all-cause and cardiovascular mortality in the general population

	Adjusted HR (95% CI), P-value
**All-cause mortality**	
Fitting by the standard linear model	1.47 (1.26, 1.70) < 0.01
Fitting by the two-piecewise linear model	
Inflection point	1.40
UAR < 1.40	0.68 (0.50, 0.93) 0.02
UAR ≥ 1.40	2.11 (1.74, 2.55) < 0.01
P for log-likelihood ratio	< 0.01
**CVD mortality**	
Fitting by the standard linear model	1.84 (1.37, 2.47) < 0.01
Fitting by the two-piecewise linear model	
Inflection point	1.88
UAR < 1.88	1.07 (0.73, 1.58) 0.73
UAR ≥ 1.88	5.21 (3.06, 8.87) < 0.01
P for log-likelihood ratio	< 0.01

Adjusted for age, gender, race, education level, marital status, PIR, BMI, smoking status, drinking status, hypertension, diabetes, CHF, CHD, HDL, LDL, TG, TC, ALT, AST, HbA1c, and eGFR.

## Abbreviations

UAR: uric acid to albumin ratio
CVD: cardiovascular disease
NHANES: national health and nutrition examination survey
PIR: family poverty income ratio
BMI: body mass index
TG: triglyceride
TC: total cholesterol
HDL-c: high density lipoprotein cholesterol
LDL-c: low density lipoprotein cholesterol
HbA1c: glycohemoglobin
CHF: congestive heart failure
CHD: coronary heart disease
eGFR: estimated glomerular filtration rate
ALT: alanine aminotransferase
AST: aspartate aminotransferase
NCHS: national center for health statistics
NDI: national death index
HR: hazard ratio
95%CI: 95% confidence interval
